# Two-Injection Start Regimen of Long-Acting Injectable Aripiprazole: Retrospective Data From a Tertiary Care Hospital in Turkey

**DOI:** 10.31083/AP44278

**Published:** 2025-06-26

**Authors:** Ayşe Nur İnci Kenar, Selin Balki Tekin

**Affiliations:** ^1^Department of Psychiatry, Faculty of Medicine, Pamukkale University, 20160 Denizli, Turkey; ^2^Clinic of Psychiatry, Denizli State Hospital, 20010 Denizli, Turkey

**Keywords:** long-acting injectable aripiprazole, aripiprazole once monthly, two-injection start regimen, schizophrenia, bipolar disorder, schizoaffective disorder

## Abstract

**Objective:**

This study aimed to evaluate the safety, tolerability, and efficacy of a newly developed two-injection start (TIS) regimen of aripiprazole once-monthly (AOM) in adult patients diagnosed with bipolar disorder, schizophrenia, or schizoaffective disorder.

**Methods:**

This retrospective study included patients diagnosed with schizophrenia, bipolar disorder, or schizoaffective disorder who were regularly followed at our clinic between January 2023 and October 2024 and initiated on the AOM treatment following the TIS regimen. Recorded data included diagnoses, sociodemographic characteristics (age, gender), concurrent psychotropic medications at the time of AOM-TIS initiation, time of hospital discharge following AOM-TIS administration, and details regarding their last AOM treatment.

**Results:**

This study included 29 patients (17 females and 12 males; mean age 36.62 ± 12.18 years) who received AOM according to the TIS regimen. AOM was administered as monotherapy in 48.2% of cases. Three patients initiated treatment directly with the AOM-TIS regimen, while nine patients did not receive any concomitant psychotropic medication. Five patients were prescribed mood stabilizers in combination with the AOM-TIS regimen. No serious adverse events, including arrhythmias, severe hyperthermia, impaired consciousness, akathisia, or allergic reactions, were reported following AOM-TIS administration.

**Conclusions:**

The AOM-TIS regimen enables the attainment of therapeutic plasma concentrations within a shortened timeframe, facilitating a more rapid clinical response. This approach may contribute to reduced healthcare costs by shortening the duration of hospitalization and enhancing patient adherence, supported by its favorable tolerability profile.

## Main Points

1. This study aims to evaluate the safety, tolerability and efficacy of a TIS regimen of long-acting injectable aripiprazole in adult patients with bipolar disorder, schizophrenia, and schizoaffective disorder.
2. With the newly developed double-onset regimen of long-acting aripiprazole, therapeutic concentrations are reached rapidly, and treatment response is achieved in a shorter timeframe.
3. The AOM-TIS regimen has the potential to reduce healthcare costs by shortening hospital stays, improving patient compliance due to its high tolerability, and preserving patient autonomy.

## 1. Introduction

It is evident that a significant proportion of individuals diagnosed with chronic psychiatric conditions, including schizophrenia and bipolar disorder, exhibit non-adherence to medication and treatment regimens. Polypharmacy may be applied in treatment-resistant patients or when switching from one antipsychotic to another, and one study found that patients with schizophrenia were using 2.0 ± 0.81 antipsychotics and taking 3.52 ± 2.55 pills per day [1, 2]. The high number of tablets taken daily may contribute to impaired long-term treatment compliance. This often results in recurrent relapses requiring hospitalization [3]. Consequently, recent studies have underscored the importance of prescribing long-acting injectable (LAI) antipsychotics from the early stages of these chronic illnesses. LAI antipsychotics could be considered as first-line treatment for patients hospitalized for first-episode psychosis, as they have proven effective in preventing relapse [4]. This approach aims to ensure continuity of treatment, reduce hospitalizations, and prevent relapses [5, 6]. However, the question of which LAI antipsychotic should be the preferred choice remains unresolved. First-generation LAI antipsychotics were introduced over 50 years ago, whereas the development of LAI formulations of second-generation agents began in the early 2000s [7]. The debate surrounding the side effects, safety profiles, and therapeutic efficacy of first- and second-generation depot injectable antipsychotics has persisted for a considerable time.

The predominance of first-generation long-acting antipsychotics in emergency settings, coupled with their propensity to induce extrapyramidal symptoms and adverse effects such as tardive dyskinesia, has contributed to a prevailing negative sentiment toward their use. In contrast, second-generation antipsychotics, owing to their distinct receptor profiles, have demonstrated a reduced risk of extrapyramidal symptoms along with a lower incidence of metabolic adverse events [8, 9]. Recently, an emerging consensus in both the literature and clinical practice suggests that second-generation LAI antipsychotics are superior and safer for both treatment and maintenance compared to first-generation agents [10, 11].

Second-generation depot antipsychotics have been shown to be effective during both the acute and maintenance phases of schizophrenia and bipolar disorder, with the potential to regulate mood, reduce side effects, and enhance quality of life and well-being [12, 13, 14, 15, 16, 17].

Aripiprazole is the first member of the dopamine partial agonist antipsychotic class, often referred to as third-generation antipsychotics. As a result, it does not cause D₂ receptor upregulation and is associated with a lower incidence of extrapyramidal symptoms. It was approved by the U.S. Food and Drug Administration for use in adult patients with schizophrenia and bipolar disorder [18]. A study investigating long-acting aripiprazole demonstrated that improvements in psychopathology and social functioning in patients with early-stage schizophrenia, as well as improvements in metabolic abnormalities in patients with chronic schizophrenia, were more pronounced with the long-acting formulation [19].

Aripiprazole once monthly (AOM) is an antipsychotic requiring a monthly parenteral dose of 400 mg, with oral aripiprazole administered during the first two weeks following injection to ensure optimal blood concentrations at treatment initiation. Since AOM is often prescribed for patients with poor adherence, managing the two-week oral supplementation phase after the first injection can be challenging. Consequently, a novel and simplified initiation strategy, designated the two-injection start (TIS) regimen, has been authorized in Europe. This regimen involves two concurrent injections at separate sites, accompanied by a single oral dose of 20 mg aripiprazole on the same day [20]. It has been demonstrated that the newly developed AOM-TIS regimen can achieve therapeutic concentrations within 24 hours, whereas the single-dose AOM initiation regimen reaches therapeutic concentrations after 14 days. This novel approach significantly reduces the time to therapeutic levels, suggesting a more rapid treatment response. A study published in 2021 comparing these two initiation regimens concluded that the TIS regimen may be preferred, given its comparable pharmacokinetic and safety profiles, along with its potential to reduce disease recurrence and enhance patient compliance [21].

To the best of our knowledge, there are only a few case reports and studies in the English literature addressing the current AOM regimen [21, 22, 23, 24, 25, 26]. Therefore, the real-world data provided by this study are valuable, as they add greater depth to the existing body of knowledge. The present study aims to evaluate the safety, tolerability, and efficacy of the TIS regimen of LAI aripiprazole in adult patients diagnosed with bipolar disorder, schizophrenia, and schizoaffective disorder, utilizing real-world data. It has been observed that the experiences reported in previous studies predominantly pertain to schizophrenia. It is anticipated that the current study will make a significant contribution to the literature by incorporating case experiences from patients with schizoaffective disorder and bipolar disorder.

## 2. Materials & Methods

This retrospective study was conducted by a trained psychiatrist who reviewed the electronic records of the hospital clinic between January 2023 and October 2024. The study comprised patients who had been attending regular follow-up sessions at the clinic and who had been administered AOM long-acting injectable antipsychotic (LAIA), with documented clinical benefit from the treatment. Patients over the age of 18 with a diagnosis of schizophrenia, bipolar disorder, or schizoaffective disorder who had initiated AOM according to the TIS regimen (two AOM injections of 400 mg each at two separate gluteal and/or deltoid injection sites, for a total of 800 mg of injectable aripiprazole, combined with a single oral dose of 20 mg aripiprazole) were included. It was initially planned to exclude patients who discontinued treatment; however, no patients met this exclusion criterion. The study population comprised 25 inpatients from our clinic and 4 patients under outpatient follow-up. Diagnoses, sociodemographic characteristics (such as age and gender), psychotropic medications used at the time of AOM-TIS regimen administration, hospital discharge times following AOM-TIS administration, and the timing of the last AOM treatment were recorded. Due to variability in the psychotropic doses used by the patients, individual dosages were not detailed for each case; instead, the effective (therapeutic) dose of each psychotropic agent was considered. Diagnoses were established by the primary psychiatrist according to the criteria specified in the Diagnostic and Statistical Manual of Mental Disorders, Fifth Edition (DSM-5) [27]. Given the retrospective design, patient hospital records and clinical file notes from regular follow-ups were analyzed to assess treatment effectiveness.

For inpatients, the clinical treatment team, consisting of the primary physician, nursing staff, and patient carer, monitored and documented treatment effectiveness and any side effects experienced during the hospitalization period following AOM-TIS administration. For outpatients, the primary physician assessed the patient at the follow-up injection appointment one month later and recorded any reported side effects. Data for this study were obtained through a retrospective analysis of these records.

### Statistical Analyses

The conformity of continuous variables to normal distribution was checked by Shapiro-Wilk’s test. Data conforming to normal distribution are shown as mean ± standard deviation (SD). Categorical variables are shown as numbers and percentages. Statistical analysis was performed with SPSS for Windows Version 27.0 (IBM Corp., Armonk, NY, USA).

## 3. Results

The present study comprised 29 patients (17 females and 12 males, mean age 36.62 ± 12.18 years, range 20 to 62 years) who were initiated on AOM according to the TIS regimen. Of the four patients who were started on the AOM-TIS regimen during outpatient follow-up, two were diagnosed with schizophrenia and the other two with bipolar disorder, manic episodes. The remaining patients were initiated on this regimen during inpatient treatment, with the diagnostic distribution of these patients presented in Table 1. According to clinical observations made by the primary treatment team, improvements in clinical symptoms were noted after the first week in one case, while improvements were observed by the fifth day in all other cases. The mean duration of hospitalisation following the administration of the AOM-TIS regimen was 21.16 ± 11.30 days, with a range of 3 to 45 days. The distribution of psychotropic drugs according to diagnosis at the time of AOM-TIS regimen application is presented in Table 2. In 48.2% of cases, patients received AOM monotherapy. While 31% of these cases received only AOM antipsychotic treatment without mood stabilisers, 17.2% received AOM antipsychotic treatment with mood stabilisers. Only AOM treatment was administered to three patients who had received no prior treatment. In six patients, the medications used before AOM treatment were discontinued after AOM-TIS regimen administration, and only AOM was continued as maintenance treatment. Five patients were prescribed mood stabilisers exclusively in conjunction with the AOM-TIS regimen (see Tables 3,4). No serious adverse effects, such as cardiac rhythm irregularity, severe fever, disturbance of consciousness, akathisia, or allergy that would prevent the continuation of treatment, were observed in any patient after administration of AOM-TIS. Treatment with AOM is ongoing in all patients.

**Table 1.** The distribution of patients’ diagnoses*.

Diagnoses	n (%)
Bipolar Disorder Depressive Episode	1 (3.4%)
Bipolar Disorder Manic Episode	8 (27.6%)
Schizoaffective Disorder	4 (13.8%)
Schizophrenia	16 (55.2%)
Total	29 (100.0%)

*Descriptive statistics were calculated.

**Table 2.** Distribution of psychotropic drugs according to diagnosis at the time of AOM-TIS regimen application*.

		Diagnoses				Total (n)
		Bipolar Disorder Depressive Episode (n)	Bipolar Disorder Manic Episode (n)	Schizoaffective Disorder (n)	Schizophrenia (n)	
Psychotropics	Lithium+Risperidone	0	2	0	0	2
	Lithium + Risperidone + Olanzapine	0	1	0	0	1
	Olanzapine	0	0	0	5	5
	Olanzapine + Amisulpride	0	0	0	2	2
	Paliperidone	0	0	0	2	2
	Paliperidone + Haloperidol	0	0	0	1	1
	Paliperidone + Olanzapine	0	0	2	1	3
	Paliperidone + Valproic Acid	0	1	1	0	2
	Risperidone + Olanzapine	0	0	0	1	1
	Valproic Acid	0	2	0	0	2
	Valproic Acid + Amisulpride	0	1	0	2	3
	Valproic Acid + Olanzapine	0	1	1	0	2
	None	1	0	0	2	3
Total		1	8	4	16	29

*Descriptive statistics were calculated. AOM, aripiprazole once-monthly; TIS, two-injection start.

**Table 3.** Distribution of psychotropic drugs used after the AOM-TIS regimen*.

Psychotropic Drugs	n (%)
AOM + AP	8 (27.6%)
AOM + AP + MS	7 (24.1%)
AOM + MS	5 (17.2%)
Only AOM	9 (31.0%)
Total	29 (100.0%)

*Descriptive statistics were calculated. AP, Antipsychotic; MS, Mood Stabilizer.

**Table 4.** **Psychotropic drugs used before and after AOM-TIS regimen**.

Cases	Before AOM-TIS Regimen	After AOM-TIS Regimen
Case 1	Olanzapine	Same treatment
Case 2	Paliperidone + Valproic Acid	Same treatment
Case 3	Paliperidone + Haloperidol	None
Case 4	Valproic Acid	Same treatment
Case 5	Valproic Acid	Same treatment
Case 6	Paliperidone + Olanzapine	Paliperidone
Case 7	Olanzapine	None
Case 8	Olanzapine	None
Case 9	Olanzapine + Amisulpride	Same treatment
Case 10	Valproic Acid + Amisulpride	Same treatment
Case 11	Paliperidone + Valproic Acid	Same treatment
Case 12	Olanzapine	None
Case 13	None	None
Case 14	Lithium + Risperidone + Olanzapine	Lithium
Case 15	Paliperidone	Same treatment
Case 16	Valproic Acid + Amisulpride	Same treatment
Case 17	Risperidone + Olanzapine	None
Case 18	Lithium+ Risperidone	Lithium
Case 19	Paliperidone + Olanzapine	Same treatment
Case 20	Paliperidone	Same treatment
Case 21	None	None
Case 22	Valproic Acid + Amisulpride	Same treatment
Case 23	Olanzapine + Amisulpride	Olanzapine
Case 24	Lithium + Risperidone	Same treatment
Case 25	Olanzapine	None
Case 26	Valproic Acid + Olanzapine	Valproic Acid
Case 27	Valproic Acid + Olanzapine	Same treatment
Case 28	Paliperidone + Olanzapine	Same treatment
Case 29	None	None

## 4. Discussion

LAIAs have been demonstrated to result in a reduced incidence of adverse effects and to be better tolerated by patients compared with oral formulations, owing to the more stable blood concentrations they provide. This has been shown to enhance treatment adherence and allow for longer dosing intervals. In addition, LAIAs may offer clinicians an advantage in the controlled management of treatment by the healthcare team [10, 28]. AOM requires oral supplementation for the first two weeks, a requirement that may present a disadvantage in patients with treatment non-compliance. The AOM-TIS regimen, a novel form of administration, has the potential to enhance adherence during the initial phase of treatment by eliminating the need for oral supplementation and may facilitate earlier discharge by reducing hospital stay [26, 29]. In this study, we sought to contribute to the extant literature by sharing our clinical experiences regarding the safety, tolerability, and efficacy of the AOM-TIS regimen in adult patients diagnosed with bipolar disorder, schizophrenia, and schizoaffective disorder. Our findings revealed a paucity of data concerning this treatment regimen in the English literature, with the exception of a small number of case reports and studies. This apparent dearth of information may be attributed to the recent introduction of the regimen, which may have led to reluctance among psychiatrists to administer a double-dose injection on the same day due to concerns regarding potential side effects. In the study by Sungur *et al*. [22], the number of cases was small and only patients with bipolar disorder manic episodes were analysed. In the present study, data on bipolar manic and depressive episodes, schizophrenia, and schizoaffective disorders were also investigated. In the study by Cuomo *et al*. [25], although the number of cases was high, only patients with a diagnosis of schizophrenia were included. Additionally, in the study by Cuomo *et al*. [25], the psychotropic drugs used before the AOM-TIS regimen and concomitantly with AOM were specified, although it was not indicated whether several of these drugs were used simultaneously or individually with AOM. In our study, the psychotropic medications used before the AOM-TIS regimen and in combination with AOM were specified separately for each case. This provides insight into which antipsychotics and/or mood stabilisers can be combined with AOM. The present study contributes to the existing literature by including 29 cases, with additional cases of schizoaffective disorder.

It is noteworthy that none of the patients in this study experienced moderate to severe side effects requiring discontinuation of treatment, as documented in the clinical notes. In two case reports using the AOM-TIS regimen in 16-year-old patients diagnosed with schizophrenia and bipolar disorder, the regimen was well tolerated and no side effects were observed [23, 24]. A case series conducted in 2024 with eight adult patients with bipolar disorder reported no serious effects other than side effects such as sedation, tachycardia, and transient fever [22]. A study investigating the experiences of 50 psychiatrists in Spain with the AOM-TIS regimen found that the majority considered the regimen to be safe and tolerable and believed it provided high patient satisfaction [26]. In a study conducted in Italy in 2023, 133 patients diagnosed with schizophrenia were initiated on the AOM-TIS regimen. No severe adverse effects, including fever, tachycardia, disorientation, rash, or edema, were observed in any patient. Only three patients experienced mild adverse effects, such as fever, sedation, and akathisia, and these patients discontinued treatment [25]. Although there is partial compatibility between our results and those reported in the literature, the absence of moderate to severe side effects in our study may be attributable to the relatively modest sample size. In our study, clinical improvement within the first week following regimen administration was deemed satisfactory and consistent with the findings of Sungur* et al*. [22]. This may also be related to the acceleration of improvement, given that the majority of cases involved the concurrent use of additional psychotropic treatments. Except for three cases, all cases involved the use of adjunctive psychotropic drugs in conjunction with the AOM-TIS regimen, with olanzapine monotherapy being the most commonly prescribed agent. It was observed that combinations of antipsychotic plus antipsychotic or antipsychotic plus mood stabiliser were used in many cases.

The selection of psychotropic drugs by clinicians evolves over time with the introduction of new options and the accumulation of clinical experience. A study conducted in the USA identified olanzapine, risperidone, and quetiapine as the most frequently prescribed antipsychotics for patients with schizophrenia in the last decade [30]. In a study involving patients initiated on the AOM-TIS regimen, the most commonly used concurrent psychotropics were olanzapine, lithium, and valproic acid, consistent with the preferences observed in our study [25].

The duration of hospitalisation varies according to the disease, individual patient characteristics, country, and healthcare system, with estimates ranging from 2 to 5 weeks in bipolar disorder and between 3 and 10 weeks in schizophrenia [31, 32]. In the present study, the mean duration of hospitalisation following the administration of the AOM-TIS regimen was determined to be three weeks. In contrast, a study conducted in Spain reported that schizophrenia patients receiving the AOM-TIS regimen had an average hospital stay of 16 days. The data from that study also demonstrated a five-day reduction in hospitalisation duration compared to the mean for patients in national psychiatric units [26]. A case series conducted in 2024 reported that the average starting day for the AOM-TIS regimen was day 5, with a total hospitalisation period of 24 days [22]. A study conducted in Turkey involving patients with schizophrenia, bipolar disorder, or schizoaffective disorder found that the average hospital stay was 30 days [33]. In 2021, a significant reduction in the mean number of hospitalisation days was observed in patients diagnosed with schizophrenia following the initiation of AOM [29]. These findings are consistent with the results of the present study and suggest that the AOM-TIS regimen may contribute to a reduction in the duration of hospitalisation. A shorter hospitalisation period may allow patients to return to real-world environments and maintain their autonomy, potentially enhancing treatment compliance and motivation. Additionally, it may result in healthcare expenditure savings for governments.

### Limitations and Future Research

A limitation of the present study is that it was based on data obtained from clinical records due to its retrospective design, which may have resulted in some data loss. However, detailed daily file records and observations were available, as the majority of cases involved inpatients. Another limitation was the small sample size, which may limit the generalisability of the results. This may also be related to the absence of serious adverse events and the fact that no patients discontinued treatment. The lack of a scale to quantify side effects and clinical improvement, which was observed during the first month, represents an additional limitation. Furthermore, the absence of a control group is an important limitation of the study. In addition, the majority of patients were receiving concomitant psychotropic agents alongside the AOM-TIS regimen, which may have limited the ability to isolate the efficacy and safety of the AOM-TIS regimen alone in the evaluation of side effects and symptomatic improvement.

Consequently, future research may benefit from a longer prospective study design incorporating a control group to compare the efficacy of the new and old initiation regimens. The inclusion of clinical scales to collect more objective data on side effects and clinical improvement would also be advantageous. Furthermore, it is important to conduct cost-effectiveness analyses comparing the AOM-TIS regimen with oral antipsychotic treatments. The recently approved bimonthly aripiprazole injection introduces a new treatment option, and future studies investigating this formulation will be necessary [18].

## 5. Conclusions

With the newly developed TIS regimen of long-acting aripiprazole, the only D₂ partial agonist LAIA option currently available, therapeutic concentrations are reached rapidly, and treatment response may be achieved in a shorter time. This could reduce healthcare costs by shortening hospital stays, improve patient compliance due to high tolerability, and help preserve patient autonomy.

## Data Availability

Data for this article is available from the corresponding author upon reasonable request.
